# Predicting Drugs Suspected of Causing Adverse Drug Reactions Using Graph Features and Attention Mechanisms

**DOI:** 10.3390/ph17070822

**Published:** 2024-06-22

**Authors:** Jinxiang Yang, Zuhai Hu, Liyuan Zhang, Bin Peng

**Affiliations:** College of Public Health, Chongqing Medical University, Chongqing 401331, China; jxyang3@stu.cqmu.edu.cn (J.Y.); 2022120774@stu.cqmu.edu.cn (Z.H.); 2022120860@stu.cqmu.edu.cn (L.Z.)

**Keywords:** adverse drug reaction, deep learning, attention mechanism, drug safety

## Abstract

Background: Adverse drug reactions (ADRs) refer to an unintended harmful reaction that occurs after the administration of a medication for therapeutic purposes, which is unrelated to the intended pharmacological action of the drug. In the United States, ADRs account for 6% of all hospital admissions annually. The cost of ADR-related illnesses in 2016 was estimated at USD 528.4 billion. Increasing the awareness of ADRs is an effective measure to prevent them. Assessing suspected drugs in adverse events helps to enhance the awareness of ADRs. Methods: In this study, a suspect drug assisted judgment model (SDAJM) is designed to identify suspected drugs in adverse events. This framework utilizes the graph isomorphism network (GIN) and an attention mechanism to extract features based on patients’ demographic information, drug information, and ADR information. Results: By comparing it with other models, the results of various tests show that this model performs well in predicting the suspected drugs in adverse reaction events. ADR signal detection was conducted on a group of cardiovascular system drugs, and case analyses were performed on two classic drugs, Mexiletine and Captopril, as well as on two classic antithyroid drugs. The results indicate that the model can accomplish the task of predicting drug ADRs. Validation using benchmark datasets from ten drug discovery domains shows that the model is applicable to classification tasks on the Tox21 and SIDER datasets. Conclusions: This study applies deep learning methods to construct the SDAJM model for three purposes: (1) identifying drugs suspected to cause adverse drug events (ADEs), (2) predicting the ADRs of drugs, and (3) other drug discovery tasks. The results indicate that this method can offer new directions for research in the field of ADRs.

## 1. Introduction

Adverse drug reactions (ADRs) refer to an unintended harmful reaction that occurs after the administration of a medication for therapeutic purposes, which is unrelated to the intended pharmacological action of the drug. Adverse drug events (ADEs) refer to any harmful medical event that may occur during the course of treatment and could be related to medication use [1]. There are numerous factors that can contribute to adverse drug reactions, including the drug dosage, modulation of targets, off-target effects, metabolic product activity, and individual genomic differences [2]. In the United States, ADRs account for 6% of all hospital admissions annually. The cost of ADR-related illnesses in 2016 was estimated at USD 528.4 billion [3,4,5]. Therefore, the prevention and control of ADRs are of significant importance.

Increasing the awareness of ADRs is an effective measure for their prevention [5]. Currently, research on adverse drug reactions primarily focuses on prevention. It can be divided into two main approaches.

- ADR Mining: Mining potential adverse reactions from post-marketing surveillance reports of drugs, such as utilizing spontaneous reporting systems (SRS) for the passive monitoring of potential adverse drug reactions [6] or employing electronic health records (EHR) [7,8], social media platform data [9,10], and other sources for the active monitoring of adverse drug reactions [11,12,13].
- Algorithm Development: Various algorithms are developed to utilize drug structural information, target information, etc., to predict potential adverse drug reactions or to forecast drug–drug interactions (DDIs).

For ADR mining, a significant amount of research utilizes databases such as the FDA Adverse Event Reporting System (FAERS) [14] and the European Medicines Agency (EMA) [15] to explore potential adverse reactions associated with individual drugs, particularly focusing on newly marketed drugs. However, such databases have certain limitations.

Firstly, they only contain data from patients who have experienced ADRs after medication use, lacking information on overall drug usage. Therefore, it is challenging to estimate the incidence rates of adverse reactions for a specific drug solely based on these databases [16].

Secondly, these databases suffer from issues like delays, biases, and underreporting. Studies indicate that the reporting of serious adverse events to FAERS may only represent 1–13% of the actual events [17].

Thirdly, the data may lack comprehensiveness and accuracy, and there could be problems like duplicate reporting. For instance, FAERS collects spontaneous reports from various populations, and reports of adverse events submitted by non-professional healthcare personnel might contain errors in judgment regarding suspected drugs and ADRs. Additionally, the content of the EMA database is complex and lacks specific ADR retrieval features, which is inconvenient for research purposes [18].

The method of using SRS to uncover ADRs is referred to as passive monitoring. The primary limitation of passive monitoring lies in the lack of overall drug usage data, which hinders many research endeavors. In contrast to passive monitoring, there is active monitoring, which involves developing systems to actively monitor overall drug usage. This enables the systematic analysis of the adverse reactions generated, such as using EHR or data from social media platforms [19]. EHR contain clinical treatment data, with each record containing not only information about drug usage and observed ADRs but also detailed demographic characteristics and medication information [20]. In unstructured EHR, ADEs are automatically detected using decision tree models and association rule methods [21], and potential ADRs are analyzed.

With the advancement of data mining technologies and the rise of machine learning and deep learning methods, particularly the development of natural language processing (NLP) techniques, active monitoring has been greatly supported. Scholars utilize NLP methods to identify ADR entities from information extracted from EHR and social media platforms. For instance, they employ recurrent neural network (RNN) frameworks to extract adverse reaction event information and demographic data from EHR and convert it into structured data [22].

Social media platforms such as Twitter, due to their large user base, serve as good sources of adverse reaction records, with adverse reaction information being well documented within them. Research has found statistically significant correlations between certain adverse drug reactions (ADRs) described in Twitter data and ADRs reported in FAERS [23], indicating that Twitter serves as a viable source of pharmacovigilance data. Research combining RNN and bi-directional long short-term memory (BiLSTM) network frameworks has developed new methods for ADR entity recognition on Twitter datasets, demonstrating strong performance [17]. The application of word embedding techniques and attention mechanisms enhances the accuracy of ADR entity recognition [24]. For instance, one study utilized a multihop self-attention mechanism (MSAM) to acquire different attention weights from various segments, thereby capturing more semantic information for active ADR monitoring [25].

One drawback of using EHR and social media data is the need for data annotation, which requires a considerable amount of work. However, the development of self-supervised learning (SSL) has simplified this task. SSL can automatically generate labels, transforming unsupervised problems into flexible, supervised ones that are feasible to address [26]. It holds great potential for applications in EHR.

For algorithm development, a considerable amount of research utilizes known adverse reaction data about drugs and integrates information such as the drug structure, target, pathway, and proteins to explore deeper relationships between drugs and adverse reactions. This is aimed at predicting potential adverse drug reactions and interactions between drugs that lead to adverse reactions. These methods play a significant role in the development of new drugs.

In the prediction of adverse drug reactions, with the application of graph neural network (GNN) and graph isomorphism network (GIN) models to assess drugs’ chemical properties, these deep learning models have demonstrated powerful performance. Consequently, such models have also begun to be utilized for adverse drug reaction prediction. Cheng et al. have developed a multi-class prediction model based on graph encoding and a self-attention mechanism. They utilize a GIN pre-trained model to extract graph features from the drug structure and employ a multi-head self-attention mechanism to extract substructure information from the drug’s molecular fingerprint. They then use a neural network for multi-class prediction at the system organ class (SOC) level, providing a tool for the identification of potential adverse reactions in early drug development [18]. Galeano et al. utilized matrix factorization to predict the interaction scores between drugs and adverse reactions [27]. Building upon their work, Zhao et al. integrated multiple information sources to predict frequencies, achieving superior performance [28].

In terms of DDI prediction, Chen et al.’s approach is based on jointly learning drug representations using both the intrinsic structural information of drugs and a knowledge graph (KG) enriched with abundant biomedical information. They designed a dual-layer cross-strategy to better integrate multimodal features and demonstrated excellent performance across multiple drug interaction datasets [29]. Lyu et al. learned multimodal representations of drugs from drug knowledge graphs (DKG) and heterogeneous features (HF). They designed a multi-channel fusion neural layer to explore the complementarity between multi-channel drug representations and achieved promising results on real-world datasets [30]. In the field of molecular design, Xiong et al. introduced a novel graph neural network called Attentive FP for molecular representation, which employs graph attention mechanisms to learn from relevant drug discovery datasets. This method demonstrates good performance across various datasets and enables the interpretability of the learned features, along with offering a visualization approach [31].

The existing methods have achieved significant progress but still have some limitations. The studies conducted by Galeano et al. and Zhao et al. utilized matrix factorization to predict drug adverse reactions (ADRs), which cannot be generalized to new drugs because, for these drugs, the information used in the models may not be available. Furthermore, determining the scope of the ADRs to be predicted for new drugs is challenging.

In clinical practice, physicians are more concerned about quickly identifying the suspected drugs causing ADRs when a patient experiences them after taking a certain medication. This study aims to leverage deep learning methods to promptly identify the responsible drugs based on the relationships between drugs and adverse reactions after the occurrence of adverse reactions. Additionally, it seeks to explore the potential ADRs of drugs.

This study primarily employs a deep learning approach to design a suspect drug assisted judgment model (SDAJM) to achieve the following three objectives.

- The model should be capable of using generic information to assess the drugs causing ADRs in ADEs. This inference must be applicable to new drugs. For instance, by inputting patient demographics, drug SMILES encoding, and ADR information, the model should be able to infer suspected drugs.
- The model should be able to learn the relationship between the chemical structure information of existing drugs and ADRs, predicting the relationships between drugs and ADRs.
- The model should be capable of extracting the chemical structure features of drugs for tasks in drug discovery, such as predicting drug activity, toxicity, and side effects.

## 2. Results and Discussion

### 2.1. Evaluation Metrics

In this study, 10-fold cross-validation is adopted to evaluate the performance of our model. Several commonly used evaluation metrics, including accuracy, precision, recall, the F1 score, and the area under the receiver operating characteristic curve (ROC-AUC), are used to assess the performance of the model. The computation of these metrics is as follows:

(1) $$Accuracy=\frac{TP+TN}{TP+TN+FP+FN}$$

(2) $$Precision=\frac{TP}{TP+FP}$$

(3) $$Recall=\frac{TP}{TP+FN}$$

(4) $$F1=\frac{2\times Precision\times Recall}{Precision+Recall}$$

where TP, TN, FP, FN, respectively, denote true positives, true negatives, false positives, and false negatives. Accuracy represents the proportion of correct predictions, Precision represents the proportion of correctly predicted positives among all predicted positives, Recall represents the proportion of correctly predicted positives among all actual positives, and the F1 score is the weighted harmonic mean of the Precision and Recall.

### 2.2. Identifying Suspected Drugs in Adverse Drug Reaction Events

#### 2.2.1. Evaluation on FAERS Dataset and JADER Dataset

Using the organized FAERS and JADER datasets, ten-fold cross-validation was performed. The SDAJM model was compared with models such as FPGNN-SDAJM, ResNet, and CNN. In FPGNN-SDAJM, drug feature extraction was conducted using the FP-GNN model, while other parts still used SDAJM’s feature extraction methods. Validation on the FAERS dataset was conducted in two ways: the random splitting of the dataset and splitting by drug. The cross-validation results are shown in the Table 1.

On the FAERS dataset, in the random split test, the SDAJM model exhibited the best average performance, but FPGNN-SDAJM achieved the highest recall. In the test split by drug, SDAJM outperformed FPGNN-SDAJM.

In the two different partitioning approaches, the model’s performance exhibits significant differences. By observing the data structure, this study suggests the following potential issues. (1) Although the model can generalize based on the similarity between drugs, this information is limited. For an ADR that has not appeared in the training set, the model may struggle to formulate accurate predictions. (2) There are instances in the data where a drug may exhibit a certain ADR but is not considered a suspected drug for this ADE, leading to confusion in the information.

In the test on the JADER dataset, SDAJM demonstrated the best overall performance, but CNN had the highest precision. In summary, the SDAJM model performed well in the tests. For the external validation and feature evaluation of the models, please refer to Supplementary Material S2.

#### 2.2.2. External Validation and Case Analysis

**Figure 1:**
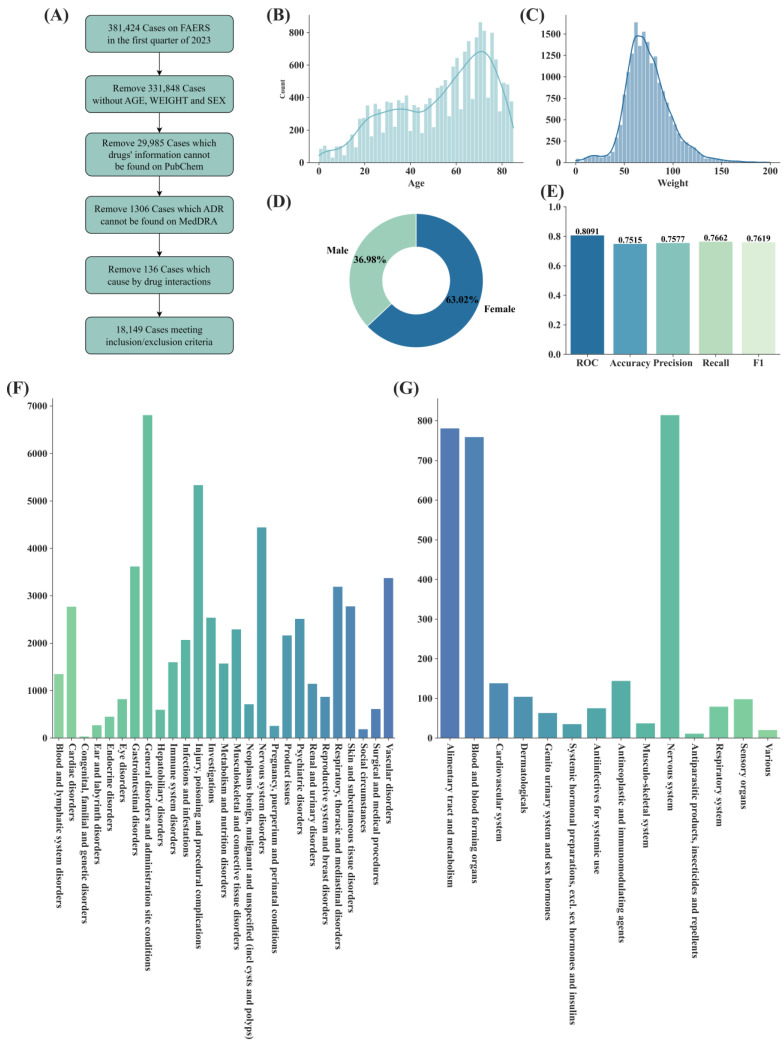
Statistical information related to the first quarter of 2023. (**A**) Data processing workflow. (**B**) The age distribution of the patients in the dataset. (**C**) The weight distribution of the patients in the dataset. (**D**) The gender distribution of the patients in the data. (**E**) The evaluation scores of the prediction results. (**F**) The number of ADEs designated within the 27 SOC categories in the dataset. (**G**) The number of drugs involved in the dataset under the ATC classification.

In this section, the SDAJM model was trained using the FAERS database from 2019 to 2022 to predict the suspected drugs causing adverse reactions in the first quarter of 2023. A total of 18,149 cases were included in the study based on previous inclusion and exclusion criteria, as illustrated in Figure 1A. Figure 1B–D depicts the distribution of the age, weight, and gender ratios in the data. Figure 1E presents the evaluation scores of the prediction results. Figure 1F illustrates the number of reports under each adverse reaction SOC category in the dataset. Figure 1G displays the classification of the drugs in the dataset based on their ATC codes.

**Figure 2:**
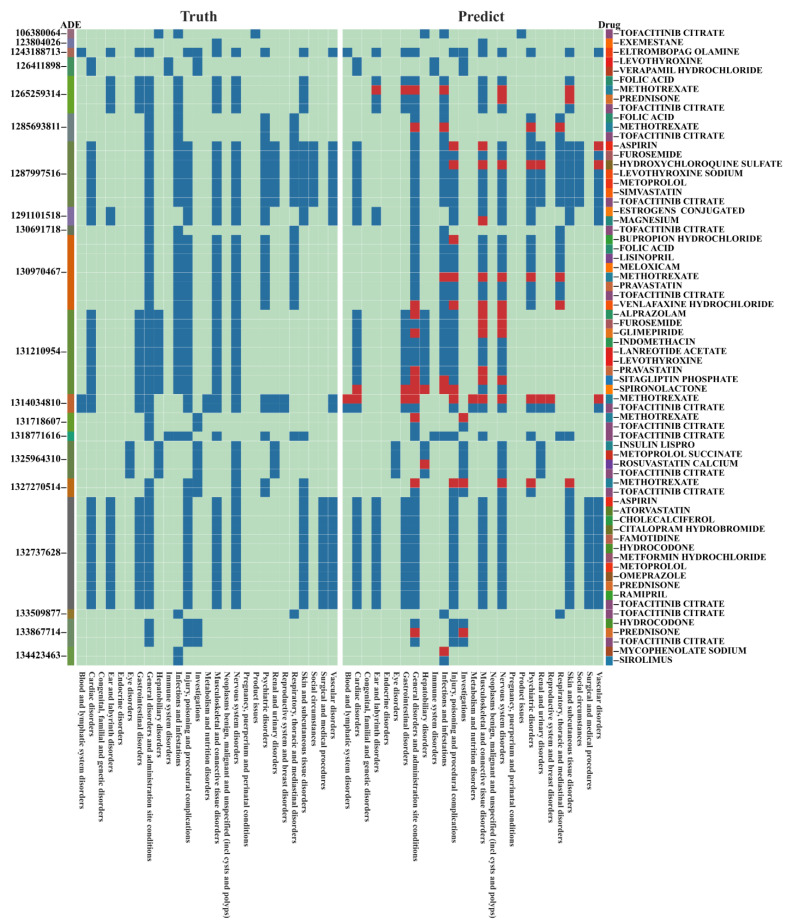
The comparison between the predictive results of SDAJM at the SOC level and the actual results in the top 20 ADEs. The left column label of the graph indicates the ADE ID, while the right column label indicates the drug name. The heatmap on the left represents the actual results, while the heatmap on the right represents the predicted results. The red areas indicate prediction errors.

Figure 2 illustrates the predictive performance of the association between the top 20 suspected drugs and ADRs (SOC level) in the medical records. Additional results can be found in Supplementary Material S3. SDAJM can infer drugs causing adverse reactions based on adverse reactions in medical records and demonstrates good performance.

To further investigate the reasonableness of the model’s predictions, this study conducted a detailed analysis of an ADE with Primary ID 100270603. The relevant information for this ADE is presented in Table 2. In this ADE, the patient used four medications, ibandronate sodium, alendronate sodium, omeprazole magnesium, and ascorbic acid, which resulted in seven ADRs: pain in the extremities, muscular weakness, a stress fracture, gait disturbance, arthralgia, low turnover osteopathy, and emotional distress. In the prediction by SDAJM, ibandronate sodium and alendronate sodium were identified as suspected drugs.

To further assess the reasonableness of the model’s predictions, this study retrieved the ADRs associated with the two drugs in question. First, the ADRs of these drugs were extracted from the SIDER database [32]. Pain in the extremities and arthralgia are ADRs associated with ibandronate sodium. Although stress fracture does not appear directly as an ADR term for this drug, it is related to terms like fracture. These ADR terms do not appear in the ADR terminology for alendronate sodium, although it includes an atypical fracture.

Further examination of the adverse reactions of these two drugs, using the results from reference.medscape.com, reveals that ibandronate sodium may cause asthenia, myalgia, and joint, bone, or muscle pain, described as severe or incapacitating. Therefore, ibandronate sodium could potentially lead to adverse reactions such as muscular weakness, gait disturbance, and arthralgia. Alendronate sodium may cause musculoskeletal pain and thus could potentially lead to adverse reactions like pain in the extremities, muscular weakness, gait disturbance, and arthralgia.

In summary, the model constructed using deep learning methods can reasonably determine the drugs causing adverse reactions.

### 2.3. ADR Signal Detection

Using the processed 2022 FAERS dataset, we conducted ADR signal detection to explore whether this method can infer the ADR information of one drug based on the relationship between the structural information of other drugs and their ADRs. This study employed two validation methods.

The first method involved training the model using a dataset that excluded cardiovascular system drugs and then conducting an ADR analysis on a set of 21 cardiovascular system drugs. These 21 drugs were associated with a total of 781 different ADRs, of which 66.72% were identified by the model as drug-related. Evidence for 58.86% of these drug–ADR associations could be directly found in the SIDER database. Two classic drugs, Mexiletine and Captopril, were selected for further investigation, with the remaining prediction results detailed in Supplementary Material S4.

The second method involved predicting the drug–ADR associations for two classic antithyroid drugs, Methimazole and Propylthiouracil.

#### 2.3.1. Investigation of ADRs to Mexiletine

Mexiletine is primarily used to suppress ventricular arrhythmias and is effective in treating peripheral neuropathy and chronic pain. Additionally, it is used in myotonic dystrophy to alleviate muscle pain and severe myotonia. Mexiletine, either alone or in combination with other antiarrhythmic drugs, is particularly effective for patients with refractory arrhythmias, eliminating spontaneous or inducible ventricular fibrillation in almost 20–50% of these patients [33].

In the processed 2022 FAERS dataset, there are only three ADEs involving Mexiletine, with only one report marking this drug as a suspect. These three reports cover a total of 11 ADRs. Due to the small number of related reports, traditional ADR signal detection methods cannot calculate the signal strength between the drug and ADRs. However, by using deep learning methods that incorporate drug structure information, it is possible to infer the relationship between the drug and ADRs based on the associations between the structures and ADRs of other drugs. Table 3 presents the ADR signal detection results for Mexiletine.

In our predictions, ADRs such as ventricular extrasystoles, congestive cardiac failure, vomiting, nausea, and headache are identified for this drug, with supporting evidence found in the SIDER database. Regarding the decreased ejection fraction, the model predicts this as an ADR for this drug. Although a search on PubMed found one report indicating a decrease in ejection fraction after using the drug, there is not enough strong evidence to confirm it as an adverse reaction to Mexiletine, so we consider it a potential adverse reaction.

For ADRs like chest discomfort, intracranial hemorrhage, and cerebral hemorrhage, the drug has corresponding ADRs at the SOC level, but there is not enough evidence to confirm these as ADRs at the PT level. For subchorionic hematoma and premature delivery, the drug does not have corresponding ADRs at the SOC level. However, the model still predicts them as potential ADRs, possibly indicating that the use of this drug during pregnancy may lead to these ADRs. Nonetheless, there is insufficient evidence to support this, and this prediction may be the result of a model error.

#### 2.3.2. Investigation of ADRs to Captopril

Captopril plays a critical role in treating hypertension, left ventricular dysfunction after myocardial infarction, and diabetic nephropathy. Its therapeutic efficacy primarily arises from its inhibition of the renin–angiotensin–aldosterone system (RAAS), making it a cornerstone in the treatment of these cardiovascular diseases. By preventing the conversion of angiotensin I to angiotensin II, Captopril effectively mitigates the pathophysiological cascade leading to hypertension and heart failure [34].

In the processed 2022 FAERS dataset, there are a total of eight ADEs involving Captopril. In three of these reports, Captopril is marked as the primary suspect drug, and in one report, it is marked as the secondary suspect drug. The reports involve a total of 29 ADRs across 13 SOC categories. Table 4 presents the ADR signal detection results for Captopril.

In the model’s predictions, ADRs such as dehydration, an aggravated condition, feeling hot, malaise, limb discomfort, altered mood, sopor, anaphylactic shock, and hypokinesia are associated with Captopril. First, after searching the SIDER database, dehydration, malaise, and hypokinesia are confirmed as ADRs for this drug. For an aggravated condition and feeling hot, there is no definitive evidence indicating these as ADRs for Captopril, but the drug has ADRs within their SOC categories, so these are considered potential ADRs in this study.

For limb discomfort, Captopril is listed as a suspected drug for the ADEs involving this reaction. While the term is not explicitly listed as an ADR in the SIDER database, the drug does have related ADRs within the musculoskeletal and connective tissue disorder SOC category, such as musculoskeletal discomfort. Therefore, this study considers limb discomfort a potential ADR. For altered mood and sopor, Captopril is a suspect drug in the relevant ADEs. The drug has some ADRs in the psychiatric disorder SOC category, such as insomnia, somnolence, and nervousness. Although these two specific reactions are not explicitly listed as ADRs for Captopril, this study considers them potential ADRs. For hypokinesia, Captopril is identified as a suspect drug in the ADEs. The drug has some related ADRs, such as bradyphrenia and muscular weakness, so hypokinesia is also considered a potential ADR.

In summary, the predictions for Captopril’s ADRs, while not all fully substantiated with evidence, are reasonably plausible. The model demonstrates its ability to identify even less frequently reported ADRs, providing valuable insights into the drug’s safety profile.

#### 2.3.3. Predicting the Drug–ADR Associations for Methimazole and Propylthiouracil

Methimazole, a thionamide medication, is crucial in treating hyperthyroidism and related conditions [35]. Propylthiouracil is an anti-thyroid drug used to manage Graves disease and hyperthyroidism [36].

This study extracted the ADRs for Methimazole and Propylthiouracil from the SIDER database, comprising a total of 48 different ADRs. The trained model was first used to predict these known drug–ADR associations, with accuracy of 84.62%. Detailed prediction results are provided in Supplementary Material S5. Further, new drug–ADR associations were constructed based on the differences in the ADRs between the two drugs, and the model’s predictions were analyzed to determine whether there was evidence supporting these associations.

For Methimazole, 16 new associations were constructed, and the model’s predictions excluded the association with dysgeusia. For Propylthiouracil, two new associations were constructed. The results and supporting evidence are presented in Table 5.

PubMed was used to search for ADRs related to Methimazole. Regarding exfoliative dermatitis, the existing literature reports that Methimazole may cause various skin-related adverse events, such as a maculopapular rash, skin pigmentation, urticaria, exfoliative dermatitis, and toxic epidermal necrolysis [37]. Regarding erythema nodosum, there is a documented case of severe erythema nodosum induced by Methimazole [38]. For skin ulcers, the literature reports a case of an acral ulcer occurring after the use of Methimazole [39], as well as a case of persistent ulcers on the lower leg after prolonged Methimazole treatment [40].

Regarding glomerulonephritis and rapidly progressive glomerulonephritis, the literature reports a case of Methimazole-induced pauci-immune glomerulonephritis [41]. For hemorrhage and hemoglobin, there is a study reporting a case of severe gastrointestinal bleeding induced by Methimazole [42]. Regarding splenomegaly, two animal experimental studies suggest that Methimazole can lead to oxidative stress and cellular damage in the spleen [43,44], and there is a reported case of a patient developing splenomegaly symptoms after using Methimazole [45].

Regarding vasculitis, a study reported an adverse reaction event of Methimazole-induced vasculitis [46]. Regarding hepatic failure, liver injury, and traumatic liver injury, existing research indicates that Methimazole can cause hepatotoxicity [44]. Some studies suggest an association between Methimazole and milder liver injury [47], while a case report documented a case of acute liver failure induced by Methimazole [48].

Regarding lung infiltration, a study reported a case of recurrent pleural effusions induced by Methimazole [49]. Antineutrophil cytoplasmic antibody positivity induced by Methimazole is relatively rare. However, there is a study reporting a case of propylthiouracil-induced drug-induced antineutrophil-cytoplasmic-antibody-positive vasculitis causing frostbite vasculitis [50]. There is insufficient evidence to suggest an association between Methimazole and interstitial lung disease.

Regarding ADR predictions related to Propylthiouracil, there is insufficient evidence to suggest an association with hypoglycemic coma. Regarding insulin autoimmune syndrome, a retrospective study reported six cases of insulin autoimmune syndrome occurring after the use of Propylthiouracil [51].

These pieces of evidence support the model’s predictions, demonstrating its ability to generate reasonable inferences based on the relationships between other drugs and adverse reactions.

### 2.4. Validation of Ten Tasks in the Field of Drug Discovery

In this section, the constructed method is evaluated using 10 public benchmark datasets in the field of drug discovery to determine its applicability to other areas of drug discovery. These 10 datasets include three physicochemical datasets, ESOL [52], FreeSolv [53], and Lipophilicity [54]; three bioactivity and biophysics datasets, MUV [55], HIV [56], and BACE [57]; and four physiology and toxicity datasets, BBBP [58], Tox21 [59], SIDER [32], and ClinTox [60,61]. The physicochemical datasets are used for regression tasks, evaluated using the RMSE, while the other datasets are used for classification tasks. Except for MUV, which is evaluated using the area under the precision recall curve (PRC-AUC), the evaluation metric used for the classification tasks is the ROC-AUC. To ensure a fair comparison, a data splitting code similar to that of Xiong et al. [31] was used, randomly dividing the datasets into training, validation, and test sets in an 8:1:1 ratio. Testing was conducted with five different random seeds, and the results are the averages of five trials. For the BACE, HIV, and BBBP datasets, scaffold splitting was additionally tested. We compared SDAJM with other advanced models (MoleculeNet [62], DMPNN (Chemprop) [63], Attentive FP [31], XGBoost [64], and FP-GNN [65]), using data from Cai et al. [65] for comparison. Table 6 displays the results of SDAJM and the other advanced models tested on the 10 public datasets.

The primary task of bioactivity and biophysics datasets is to predict the biological activity of small molecules against given targets, which is significant for new drug development. Unfortunately, SDAJM did not achieve the best performance in these three tasks. In the random split testing on the HIV bioactivity dataset, SDAJM achieved second place, but it only reached third place in the scaffold splitting task. In the multi-class task of MUV, SDAJM performed second-best. Despite not achieving the best performance, SDAJM still exhibited good performance on these three datasets.

The main task of physiology and toxicity datasets is to predict the effects of molecules on the body, which is crucial for early drug development to exclude inappropriate molecules. The tasks include predicting blood–brain barrier penetration in BBBP, predicting side effects in SIDER, and predicting toxicity in Tox21 and ClinTox. The best performance was achieved on Tox21. In both the random split and scaffold split tasks of BBBP, as well as in ClinTox, moderate performance was observed with SDAJM. However, the best performance was achieved on the SIDER dataset, significantly exceeding that of the other models. The tasks of physicochemical datasets are to predict the physicochemical properties of molecules, which can reflect their pharmacokinetic stages in vivo. Therefore, accurately predicting the physicochemical properties of molecules is helpful for drug discovery and development.

SDAJM performed poorly in the tasks of FreeSolv, ESOL, and Lipophilicity, showing a considerable gap compared to the advanced models. Compared to its performance in classification tasks, SDAJM exhibits poorer performance in handling regression tasks compared to other models. After comparing the architectures of the different models, it was found that SDAJM utilizes more generic features for drug feature extraction, while the other models extract a wider range of features. For example, besides utilizing graph features, FP-GNN also employs molecular fingerprints such as MACCS, PubChem, and Pharmacophore ErG. However, incorporating more features increases the training time. SDAJM strikes a more cautious balance between drug feature extraction and ADR feature extraction. Consequently, its performance in regression tasks is inferior to that of other models.

In conclusion, the drug feature processing module of SDAJM has great potential in handling classification tasks, but its performance in handling regression tasks is relatively poor. However, this still suggests the potential application of SDAJM’s drug feature extraction method in other drug discovery fields.

## 3. Materials and Methods

### 3.1. Datasets

To evaluate the model’s performance, a dataset was constructed using data from the FAERS database for experimentation. Data from 2019 to 2022 were included, where demographic information (age, weight, sex) was not null and was reported by professionals, and the main component information of the drug could be retrieved from PubChem (https://pubchem.ncbi.nlm.nih.gov/). ADR information was standardized using MedDRA 23.0, where terms were unified to the PT level. ADR terms in the FAERS database were mostly at the PT level of the MedDRA terminology, with a few cases at the lowest level term (LLT) level. Demographic information was standardized by normalizing the units of age and weight and encoding sex, where males were represented as 0 and females as 1. For the standardization of drug information, using the Python package PubChemPy (1.0.4) provided by PubChem, drugs’ CID and SMILES were retrieved based on the active substances of the drugs provided for each ADE.

Additionally, to verify that our model could operate on different databases, the Japanese Adverse Drug Event Report (JADER) database was also curated for experimentation [66]. The handling of demographic information and ADR processing was similar to that for the previous dataset. For the Japanese drug data, the API provided by KEGG (https://www.kegg.jp/kegg/rest/) was utilized for retrieval, connecting to PubChem to obtain the drug’s CID and SMILES data. The dataset information after information extraction is shown in Table 7.

### 3.2. Framework of SDAJM

**Figure 3:**
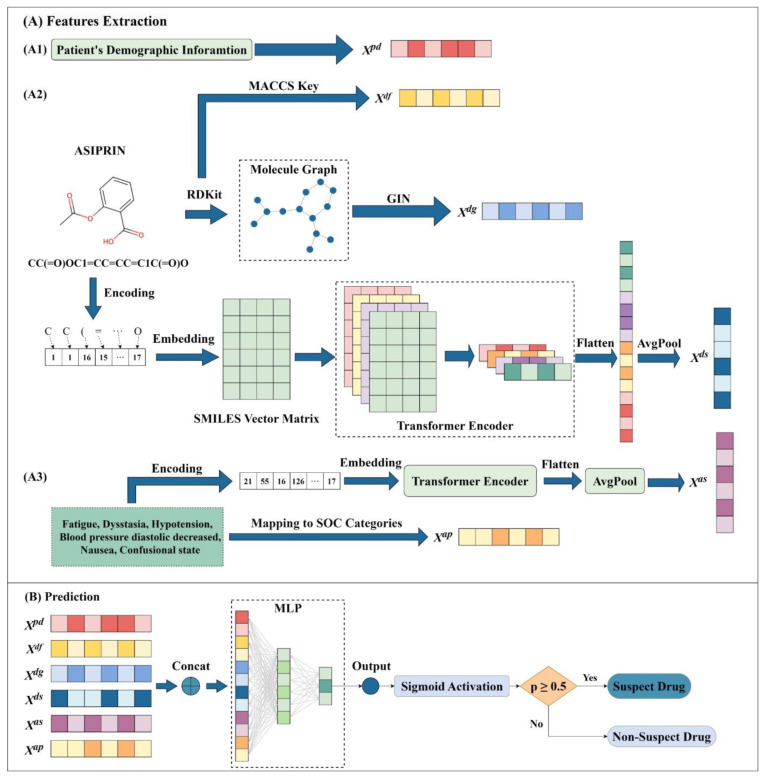
The flowchart of SDAJM. (**A**) Feature extraction of SDAJM. (**A1**) Patients’ demographic information is encoded to generate features. (**A2**) Extraction of molecular fingerprint features using SMILES: graph feature is extracted using the GIN model; SMILES sequence feature is extracted using attention mechanisms. (**A3**) Text features of ADRs are extracted using attention mechanisms. SOC category feature is extracted by encoding ADR terms by mapping them to SOC categories. (**B**) Prediction of SDAJM. All extracted features are combined and fed into an MLP framework for prediction.

The system architecture of SDAJM is shown in Figure 3. In the feature extraction phase, demographic features are encoded to generate features. Drug information is encoded using the simplified molecular-input line entry system (SMILES) to extract molecular fingerprint features; graph features are extracted using the GIN model; and the sequence features of SMILES are extracted using attention mechanisms. ADR information is extracted through two steps: firstly, semantic features are extracted using attention mechanisms; secondly, the ADR terms are mapped to the SOC categories to encode and extract the SOC category features. In the prediction phase, all extracted features are combined and fed into a multi-layer perceptron (MLP) framework for prediction. Supplementary Material S1 provides the detailed implementation methods and formula derivations.

### 3.3. Extraction of Demographic Features

To extract demographic features, information such as the patients’ age, sex, and weight data were extracted. The patients’ weight and age were standardized, while gender information was encoded, assigning 0 for male and 1 for female. After concatenating these features, the patients’ demographic features, denoted as $X^{pd}$, were derived.

### 3.4. Extraction of Drug Features

Each drug is represented using SMILES, and features are extracted based on SMILES. SMILES consist of a continuous series of letters, which are converted using the vertical first traversal tree algorithm to represent the chemical structure. In SMILES’ basic rules, hydrogen atoms are omitted, and aromatic ring structures are represented by chain opening or directly in Kekaula form. During expression, the atoms at the ends of split bonds are labeled with numbers, and side chains are written in parentheses [67]. For drug features, they are represented in three parts: molecular fingerprint features containing the drug substructure information, graph features containing the drug’s chemical structure and atomic information, and SMILES sequence features containing the SMILES sequence information.

#### 3.4.1. Extraction of Molecular Fingerprint Features for Drugs

The Molecular ACCess System (MACCS) is a molecular substructure-based fingerprint, which employs a set of predefined binary key–value pairs to represent a molecule [68]. Specifically, for each predefined substructure, if the molecule contains this substructure, the corresponding key-value pair is set to 1; otherwise, it is set to 0. MACCS keys have two lengths, 166 bits and 960 bits, depending on the number of substructure types. The 166-bit form is most commonly used. In this study, the SMILES is converted into a 167-dimensional binary vector using the Python package RDKit, resulting in feature $X^{df}$.

#### 3.4.2. Extraction of Drug Graph Features

Before extracting the graph features for drugs, it is necessary to convert the drug into an undirected graph based on the drug’s SMILES representation. In the undirected graph, each node corresponds to the information of each atomic node in the drug structure. Inspired by MUFFIN [29] and iADRGSE [18], the chirality information of the structural atoms and the types and directions of chemical bonds are extracted from the drug and assigned to the corresponding nodes.

We further perform feature extraction using the GIN model. Based on aggregating the node features in the graph according to the structure of edges, the GIN model introduces the requirement of isomorphism, meaning that the graph features after processing isomorphic graphs should be the same, while the graph features after processing non-isomorphic graphs should be different. Leveraging this characteristic, the GIN model has demonstrated strong capabilities in the field of graph neural networks [69,70,71].

The feature extraction process of the GIN model consists of two stages: information aggregation and readout. In the information aggregation stage, information from neighboring nodes is acquired, and the current node is updated using an aggregation function. The aggregation function is represented by an MLP, which theoretically can simulate the combination of functions. For the readout stage, the aim is to extract graph features that primarily represent the shape of the drug’s chemical structure. With this method, drug graph feature $X^{dg}$ is obtained.

#### 3.4.3. Extraction of SMILES Sequence Features

The SMILES encoding is treated as a document, where each symbol is considered as an individual word. It is assumed that the arrangement of SMILES also represents part of the drug’s features. Therefore, the Transformer encoder is employed for feature extraction [72]. To ensure the applicability of this method to all drugs, SMILES element statistics are obtained based on PubChem’s drugs, and a SMILES element vocabulary for encoding is constructed. With this method, SMILES sequence feature $X^{ds}$ is obtained.

### 3.5. Extraction of ADR Features

#### 3.5.1. Extraction of SOC Category Features

The ADR terms of each medical record are mapped to the SOC level. If there are ADRs corresponding to the SOC, they are encoded as 1; otherwise, they are encoded as 0. Through this encoding method, a 27-dimensional vector $X^{as}$ representing the adverse reaction encoding features is obtained.

#### 3.5.2. Extraction of ADR Semantic Features

The number of ADR terms at the PT level varies for each patient, and there are many PT-level ADR terms. Using one-hot encoding to generate features results in sparsity, making it difficult to extract features. Therefore, the PT-level ADRs involved for each patient are classified according to their primary SOC. Subsequently, an embedding module is used to assign a trainable embedding to each ADR. Features are extracted through an attention mechanism, and ADR features are obtained through an adaptive average pooling layer. With this method, ADR semantic feature $X^{ap}$ is obtained.

### 3.6. Prediction

A prediction model based on an MLP framework is constructed to determine whether the drug is a suspected causative agent of adverse reactions, utilizing the features extracted from various sources. Data for each part are obtained as follows: demographic feature $X^{pd}$, MACCS molecular fingerprint feature $X^{df}$, molecular graph feature $X^{dg}$, SMILES sequence feature $X^{ds}$, SOC category feature $X^{as}$, and ADR semantic feature $X^{ap}$. The extracted features are concatenated for classification by SDAJM. A three-layer MLP classifier was constructed with ReLU activation functions (${FC}_{relu}$) and batch normalization (BN) [73]. A fully connected layer was added at the end for aggregation. Dropout layers were included after the first two ReLU activation functions to prevent overfitting, implementing the final prediction through this module.

### 3.7. Optimization of SDAJM

Hyperparameter optimization plays a crucial role in optimizing neural network models [74,75]. For the optimization of SDAJM, our main optimization targets include the batch size, the number of attention heads in multi-head attention, the dropout rate for the GIN and predictor’s dropout layers, the learning rate, and the regularization strength. To optimize these hyperparameters, the Bayesian optimization algorithm is employed. The details of the relevant results are given in Supplementary Material S6.

### 3.8. Training Equipment and Time Consumption

The model training was conducted using the supercomputing platform at Chongqing Medical University. Details regarding the resources utilized and the time consumed for training can be found in Supplementary Material S7.

## 4. Conclusions

This study applied deep learning methods to construct an SDAJM model for three purposes: (1) identifying suspected drugs in ADEs, (2) detecting drugs’ adverse reaction signals, and (3) other drug discovery tasks. For Task 1, this study found that the use of deep learning methods can achieve relatively accurate predictions. The model only requires easily accessible information such as patient demographics, the drug’s SMILES information, and the ADR details, making it user-friendly for both healthcare professionals and consumers. The study investigated the reasonableness of the model’s predictions through specific cases and found that the predictions were indeed reasonable based on the evidence retrieved. For Task 2, the study conducted ADR signal detection for Mexiletine and Captopril. The evidence shows that this method can not only detect less frequent ADRs but also make reasonable predictions. For Task 3, comparisons with other models indicated that the deep-learning-based model developed in this study can be applied to other tasks in the field of drug discovery. In summary, the application of deep learning methods can provide new directions for research in the field of ADRs.

### Limitations and Future Prospects

Although SDAJM performs well across various tasks, it still has the following shortcomings.

- In the training dataset, although some drugs have known associations with the occurring ADRs, they may not necessarily be the suspect drugs causing these ADRs in the ADEs. This phenomenon may lead to confusion in the model’s understanding of the associations between the drugs and ADRs.
- While the model considers as much information as possible within a single ADE, it does not incorporate information from other drugs. This limitation is due to the current data structure. Future research should consider how to integrate additional information from other drugs and ADRs. Moreover, it would be beneficial to include data on the treatment duration and drug indications.
- The model balances drug feature extraction and ADR feature extraction using validated, ADR-related effective features. However, this results in reduced performance in drug feature extraction for other drug discovery tasks, especially in handling regression tasks. Future considerations should focus on adding more features or adopting more effective extraction methods, such as using pre-trained models, without significantly increasing the training time.

In future work, we aim to further integrate information within ADEs, such as using knowledge graph approaches to modify the data structure. Additionally, we will explore whether the constructed model can be extended to more fields, such as the detection of and medication for COVID-19.

## Figures and Tables

**Table 1:** The 10-fold cross-validation results on the FAERS dataset and JADER dataset.

Dataset	Split Type	Model	ROC-AUC	Accuracy	Precision	Recall	F1
FAERS	Random	SDAJM	**0.8937**	**0.8165**	**0.8403**	**0.8406**	**0.8404**
	Random	FPGNN-SDAJM	0.8730	0.8078	0.8195	0.8445	0.8318
	Random	ResNet	0.8609	0.7860	0.8150	0.8246	0.8197
	Random	CNN	0.8522	0.7820	0.7908	0.8310	0.8104
	Drug	SDAJM	0.8071	0.7404	0.8185	0.7492	0.7823
	Drug	FPGNN-SDAJM	0.7875	0.7253	0.7787	0.7427	0.7603
JADER	Random	SDAJM	**0.8462**	**0.7990**	**0.7086**	**0.7325**	**0.7203**
	Random	FPGNN-SDAJM	0.8335	0.7818	0.7508	0.5877	0.6593
	Random	ResNet	0.8030	0.7593	0.7130	0.5206	0.6018
	Random	CNN	0.8130	0.7554	0.7747	0.4230	0.5472

Note: Ten-fold cross-validation was conducted using the FAERS and JADER datasets. For the FAERS dataset, two methods were used: “random”, which randomly partitioned the dataset, and “drug”, which partitioned the dataset based on different drugs. SDAJM was compared with models such as FPGNN-SDAJM, ResNet, and CNN. In FPGNN-SDAJM, the drug feature extraction module of SDAJM was replaced with FPGNN. Bold font illustrates the models that outperformed all other models.

**Table 2:** The prediction results of SDAJM on an ADE.

Primary ID: 100270603	Age (Years): 63	Weight (kg): 47.67	Sex: Female
Drugs	Ibandronate Sodium Alendronate Sodium Omeprazole Magnesium Ascorbic Acid		
ADRs	Pain in extremities Muscular weakness Stress fracture Gait disturbance Arthralgia Low turnover osteopathy Emotional distress		
Prediction	Ibandronate sodium and alendronate sodium are suspected drugs		
Evidence	Ibandronate sodium may cause asthenia; myalgia; joint, bone, or muscle pain, described as severe or incapacitating; atypical femoral shaft fractures resulting from low energy or low trauma; osteonecrosis of the jaw and other oro-facial sites, including the external auditory canal. * Alendronate sodium may cause musculoskeletal pain. *		

Note: The results of a model-predicted ADE case. “Primary ID” represents the case number. “Drugs” provides information about the drugs used by the patient. “ADRs” provides information about the ADRs experienced by the patient. “Prediction” indicates the model’s prediction results. “Evidence” shows the relevant ADR information retrieved for the two drugs involved. * The evidence is derived from reference.medscape.com.

**Table 3:** ADR signal detection results for Mexiletine.

CID	Drug	SOC	PT	Prediction	Evidence
4178	Mexiletine	Cardiac disorders	Ventricular extrasystoles	Yes	SIDER
			Congestive cardiac failure	Yes	SIDER
		Gastrointestinal disorders	Vomiting	Yes	SIDER
			Nausea	Yes	SIDER
		General disorders and administration site cond	Chest discomfort	Yes	Unconfirmed
		Injury, poisoning and procedural complications	Maternal exposure during pregnancy	No	None
		Investigations	Decreased ejection fraction	Yes	PMID: 17392676
		Nervous system disorders	Headache	Yes	SIDER
			Intracranial hemorrhage	Yes	Unconfirmed
			Cerebral hemorrhage	Yes	Unconfirmed
		Pregnancy, puerperium and perinatal conditions	Subchorionic hematoma	Yes	Unconfirmed
			Premature delivery	Yes	Unconfirmed

Note: The associations predicted by the model between Mexiletine and ADRs are as follows. “CID” represents the PubChem CID for Mexiletine. “Prediction” indicates the model’s prediction result; if it is “Yes”, the model indicates that there is a potential association between Mexiletine and the ADR, and if it is “No”, there is no association. “Evidence” provides the retrieved evidence of the association between the drug and the ADRs.

**Table 4:** ADR signal detection results for Captopril.

CID	Drug	SOC	PT	Evidence
44093	Captopril	Metabolism and nutrition disorders	Dehydration	SIDER
		General disorders and administration site conditions	Aggravated condition	Unconfirmed
			Feeling hot	Unconfirmed
			Malaise	SIDER
		Musculoskeletal and connective tissue disorders	Limb discomfort	Unconfirmed
		Psychiatric disorders	Altered mood	Unconfirmed
			Sopor	Unconfirmed
		Immune system disorders	Anaphylactic shock	SIDER
		Nervous system disorders	Hypokinesia	Unconfirmed

Note: The associations predicted by the model between Captopril and ADRs. “CID” represents the PubChem CID for Captopril. “Evidence” provides the retrieved evidence of the association between the drug and the ADRs.

**Table 5:** The prediction results and supporting evidence for Methimazole and Propylthiouracil.

Drug	PT	Evidence
Methimazole	Exfoliative dermatitis	PMID: 15745981
	Erythema nodosum	PMID: 28725155
	Glomerulonephritis	PMID: 30214651
	Hemorrhage	PMID: 21114679
	Hemoglobin	PMID: 21114679
	Skin ulcer	PMID: 9213194, PMID: 8548997
	Splenomegaly	PMID: 21314467, PMID: 19775732, PMID: 23263868
	Vasculitis	PMID: 29760925
	Hepatic failure	PMID: 19775732, PMID: 25156887, PMID: 2271514
	Liver injury	PMID: 19775732, PMID: 25156887
	Traumatic liver injury	PMID: 19775732, PMID: 25156887
	Interstitial lung disease	Unconfirmed
	Rapidly progressive glomerulonephritis	PMID: 30214651
	Lung infiltration	PMID: 31467736
	Antineutrophil cytoplasmic antibody positivity	PMID: 27749745
Propylthiouracil	Hypoglycemic coma	Unconfirmed
	Insulin autoimmune syndrome	PMID: 26315093

Note: The results of the model-predicted associations between the artificially constructed drug and ADRs for Methimazole and Propylthiouracil are as follows. “Evidence” provides the retrieved evidence of the association between the drug and the ADRs.

**Table 6:** The performance of SDAJM on the 10 benchmark datasets.

Dataset	Split Type	Metric	MoleculeNet (Graph)	Chemprop (Optimized)	Attentive FP	XGBoost	FP-GNN	SDAJM
BACE	random	ROC		**0.898**	0.876	0.889	0.881	0.883
	scaffold	ROC	0.806 (Weave)	0.857	0.850		0.860	0.849
HIV	random	ROC		**0.827**	0.822	0.816	0.825	0.826
	scaffold	ROC	0.763 (GC)	0.794	**0.832**		0.824	0.812
MUV	random	PRC	**0.109 (Weave)**	0.053	0.038	0.068	0.09	0.093
Tox21	random	ROC	0.829 (GC)	0.854	0.852	0.836	0.815	**0.873**
BBBP	random	ROC		0.917	0.887	0.926	**0.935**	0.918
	scaffold	ROC	0.690 (GC)	0.886			**0.916**	0.911
ClinTox	random	ROC	0.832 (Weave)	0.897	0.904	**0.911**	0.840	0.841
SIDER	random	ROC-	0.638 (GC)	0.658	0.623	0.642	0.661	**0.779**
FreeSolv	random	RMSE	1.150 (MPNN)	1.009	1.091	1.025	**0.905**	1.022
ESOL	random	RMSE	0.580 (MPNN)	0.587	0.587	**0.582**	0.675	0.830
Lipophilicity	random	RMSE	0.655 (GC)	0.563	**0.553**	0.574	0.625	0.655

Note: Each dataset was split into training, validation, and test sets using the corresponding data split codes from published studies. The SDAJM models used the same dataset and data split method to fairly compare them with the MoleculeNet, Chemprop, Attentive FP, FP-GNN, and XGBoost models. Bold font illustrates the models that outperformed all other models. The values of the starred models are taken from Cai et al. [65].

**Table 7:** Information about FAERS dataset and JADER dataset.

Dataset	Number of Reports	Number of Drugs	Number of ADRs (PT)	Number of Suspect Drug Labels (Processed)	Number of Non-Suspect Drug Labels (Processed)
FAERS	206,855	3012	9315	765,161	552,044
JADER	53,528	1407	3153	146,506	265,914

Note. Dataset-related information: “Number of reports” denotes the remaining number of ADEs after processing. “Number of drugs” denotes the number of drugs in the dataset. “Number of ADRs (PT)” denotes the number of unique ADRs at the PT level in the dataset. “Number of suspect drug labels (processed)” denotes the number of labels referring to suspect drugs after processing the dataset. “Number of non-suspect drug labels (processed)” denotes the number of labels referring to non-suspect drugs after processing the dataset.

## Data Availability

The full datasets and source code for SDAJM are available on GitHub at https://github.com/ThearyYang/SDAJM (accessed on 30 May 2024).

## Supplementary Materials

The following supporting information can be downloaded at: https://www.mdpi.com/article/10.3390/ph17070822/s1, S1: Modeling; S2: Extrapolation testing and feature evaluation; S3: ADE information; S4: ADR signal detection of 21 cardiovascular system drugs; S5: The predicting results of Methimazole and Propylthiouracil; S6: Hyperparameter optimization results of 50 searches by python package hyperopt; S7: Hyperparameter optimization.
