# Supplementary material for: Predicting Drugs Suspected of Causing Adverse Drug Reactions Using Graph Features and Attention Mechanisms
Source: Pharmaceuticals (Basel). 2024 Jun 22;17(7):822. doi: 10.3390/ph17070822 (PMC11279999; doi:10.3390/ph17070822)
Supplement: Supplementary file 1 [file pharmaceuticals-17-00822-s001.zip › S1. Modeling.pdf]

Supplementary Material S1.

Modeling

1 Problem formulation

Our core work involves predicting one or more suspicious drugs that may have caused one or more adverse reactions in a patient who has experienced adverse reactions. Let P represent an information set for an ADE, where an ADE includes patient information set $I,$ drugs information set D, and ADRs information set A. For an ADE involving the use of n drugs and resulting in m ADRs, it can be represented as $P_{\{I,D,A\}}$, where $D = \{d_i|1 \leq i \leq n\}$, $A = \{adr_j|1 \leq j \leq m\}$. In this ADE, our prediction target is the label l for each drug with respect to each ADRs. That is, for each drug d_i and ADR adr_j, d_i needed to be determined whether it caused adr_j. If d_i caused adr_j, we assign $l_{d_i,adr_j} = 1$, otherwise, $l_{d_i,adr_j} = 0$. Let L represent the labels for all combinations of drugs and adverse reactions in the ADE, we can establish a mapping as $F: P_{\{I,D,A\}} \rightarrow L$, where $L = \{l_{d_i,adr_j}|1 \leq i \leq n, 1 \leq j \leq m\}$.

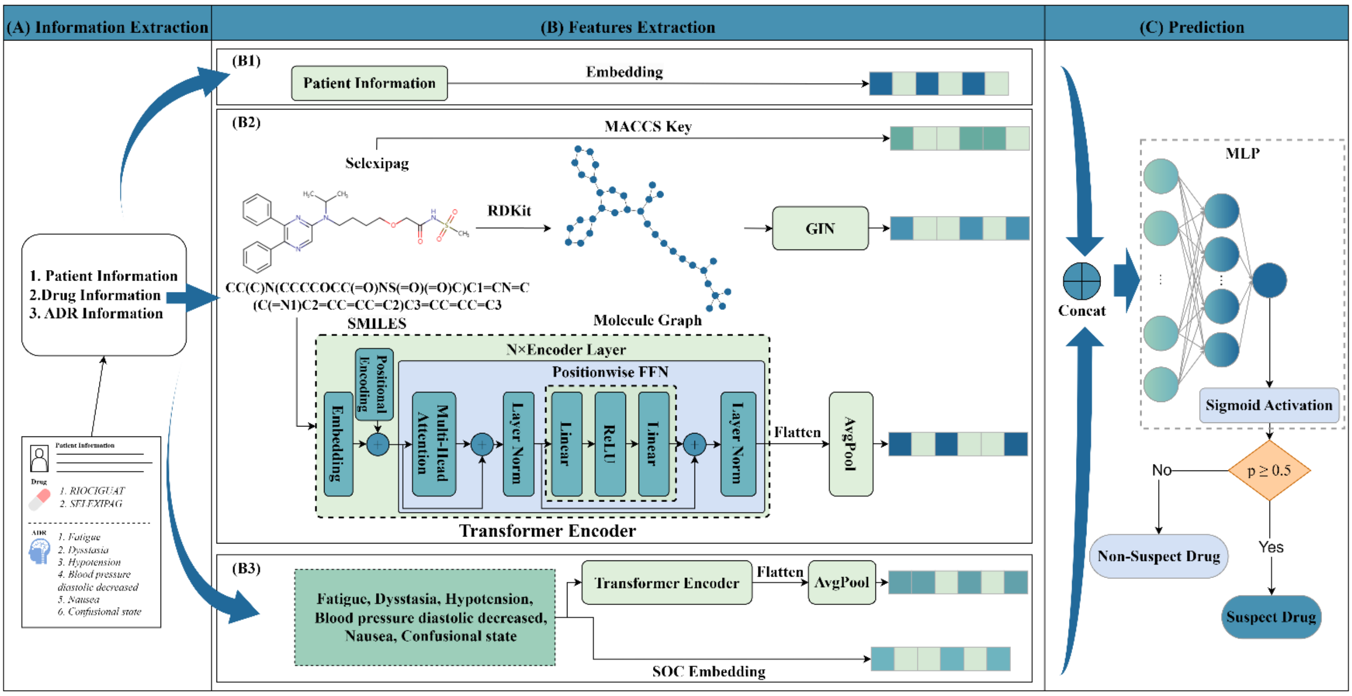

Figure S1. A flowchart of ADRGW. (A) Information extraction of ADRGW. To extract patient's personal information, drug information, and adverse reaction information from the ADE and standardize them. Drug information is obtained by extracting drug chemical structure information from active ingredients. (B) Features Extraction of ADRGW. (B1) Personal information is encoded to generate features. (B2) Extracting molecular fingerprint features using SMILES, graph features are extracted using the GIN model, SMILES text features are extracted using attention mechanisms. (B3) Text features of ADRs are extracted using attention mechanisms. SOC category features are extracted by encoding ADR terms

through mapping them to SOC categories. (C) Prediction of ADRGW. All extracted features are combined and fed into an MLP framework for prediction.

2 Framework of SDAJM

The system architecture of SDAJM is shown in **Figure S1**. In the feature extraction phase, demographic features are encoded to generate features. Drug information is encoded using the simplified molecular-input line entry system (SMILES) encoding to extract molecular fingerprint features, graph features are extracted using the GIN model, sequence features of SMILES are extracted using attention mechanisms. ADR information is extracted through two steps: firstly, semantic features are extracted using attention mechanisms; secondly, the ADRs terms are mapped to SOC categories to encode and extract SOC category features. In the prediction phase, all extracted features are combined and fed into an MLP framework for prediction.

3 Features extraction

3.1 Extraction of demographic features

To extract demographic features, information such as the patient's age, sex and weight was extracted. Patient weight and age were standardized, while gender information was encoded, assigning 0 for male and 1 for female. After concatenating these features, the patient's demographic features, denoted as X^{pd}, were derived.

3.2 Extraction of drug feature

For each drug, it is represented using SMILES, and features are extracted based on SMILES. SMILES consist of a continuous series of letters and are converted using the vertical first traversal tree algorithm to represent the chemical structure. In SMILES basic rules, hydrogen atoms are omitted, aromatic ring structures are represented by chain opening or directly by Kekule form. When expressing, atoms at the ends of split bonds are labeled with numbers, and side chains are written in parentheses [1]. For drug features, they are represented in three parts: molecular fingerprint features containing drug

substructure information, graph features containing drug chemical structure and atomic information, and SMILES sequence features containing SMILES sequence information.

(A) Extraction of molecular fingerprint feature for drug.

Molecular ACCess System (MACCS) is a molecular substructure-based fingerprint, which employs a set of predefined binary key-value pairs to represent a molecule [2]. Specifically, for each predefined substructure, if the molecule contains this substructure, the corresponding key-value pair is set to 1; otherwise, it is set to 0. MACCS keys come in two lengths: 166 bits and 960 bits, depending on the number of substructure types. The 166-bit form is commonly used. In this study, SMILES is converted to a 167-dimensional binary vector using the Python package RDKit, resulting in feature X^{df}.

(B) Extraction of drug graph feature

Before extracting graph features for drugs, it is necessary to convert the drug into an undirected graph based on the drug's SMILES representation. In the undirected graph, each node corresponds to the information of each atomic node in the drug structure. Inspired by MUFFIN [3] and iADRGSE [4], the chirality information of the structural atoms and the types and directions of chemical bonds are extracted from the drug and assigned to the corresponding nodes.

We further perform feature extraction using the GIN model. Based on aggregating node features in the graph according to the structure of edges, the GIN model introduces the requirement of isomorphism, meaning that the graph features after processing isomorphic graphs should be the same, while the graph features after processing non-isomorphic graphs should be different. Leveraging this characteristic, the GIN model has demonstrated strong capabilities in the field of graph neural networks [5-7].

The feature extraction process of the GIN model consists of two stages: information aggregation and readout. In the information aggregation stage, information from neighboring nodes is acquired, and the current node is updated using an aggregation function. The aggregation function is represented by a multi-layer perceptron (MLP), which theoretically can simulate the combination of functions. The node update method is expressed as follows:

$$h_v^{(k)} = MLP^{(k)}\left(\left(1+\epsilon^{(k)}\right)\cdot h_v^{(k-1)} + \sum_{u\in N(v)} h_u^{(k-1)}\right) \tag{S1}$$

where $h_v^{(k)}$ represents the information of node v at time k, $\epsilon^{(k)}$ denotes the weight for retaining the information of node v from the previous time step at time k, and $N(v)$ denotes the set of neighboring nodes of node v.

For the readout stage, the aim is to extract graph features that primarily represent the shape of the drug's chemical structure. The information of the graph is extracted using max pooling, expressed as:

$$X^{dg} = CONCAT\left(MaxPooling\left(h_v^{(k)} | v \in G\right) | k = 0,1,\cdots,K\right) \tag{S2}$$

(C) Extraction of SMILES sequence feature

The SMILES encoding is treated as a document, where each symbol is considered as an individual word. It is assumed that the arrangement of SMILES also represents part of the drug's features. Therefore, the Transformer Encoder is employed for feature extraction [8]. To ensure the applicability of this method to all drugs, SMILES element statistics are performed based on PubChem's drugs, and a SMILES element vocabulary for encoding is constructed. Let the length of this vocabulary be denoted as s.

a. SMILES encoding and vector matrix generation

For a SMILES sequence of drug d with length n, after encoding with the vocabulary, we obtain a vector $x_{1:n} = [x_1, x_2, \cdots, x_n], x_i \in \mathbb{R}^s$. This encoded sequence is then passed through a word embedding layer. This module provides an embedding matrix $W_e \in \mathbb{R}^{d_{model} \times s}$, where d_{model} represents the size of the Transformer Encoder hidden layer and also the dimensionality of the embedding vectors. This matrix provides a dense, learnable embedding representation for each element in the vocabulary. For each position x_i in the encoded SMILES sequence, it can be mapped to a d_{model}-dimensional vector e_i, the embedding vector $e_i = W_e x_i$.

b. Positional Encoding

The Transformer utilizes positional encoding techniques to enable the model to leverage the positional information of input sequences. It generates positional encoding matrices using sinusoidal and cosine functions with different frequencies:

$$PE_{(i,2j)} = \sin\left(\frac{i}{10000^{\frac{2j}{d_{model}}}}\right) \tag{S3}$$

$$PE_{(i,2j+1)} = \cos\left(\frac{i}{10000^{\frac{2j}{d_{model}}}}\right) \tag{S4}$$

where i represents the position and j represents the dimension.

c. Attention Mechanism

For the representation matrix $X \in \mathbb{R}^{n \times d_{model}}$ of a drug's SMILES sequence, three linear transformation matrices $W_Q, W_K, W_V \in \mathbb{R}^{d_{model} \times d_{model}}$ are used to perform linear transformations, resulting in three new vector matrices: Query Q, Key K, and Value V.

$$Q|K|V = XW_Q|XW_K|XW_V \tag{S5}$$

By computing the three new vector matrices, the attention distribution $A \in \mathbb{R}^{n \times n}$ can be calculated. For the element $A_{i,j}$ in the i-th row and j-th column of this distribution, it represents the attention weight of the i-th element in the sequence at the j-th position. Its calculation is as follows:

$$A_{i,j} = Softmax\left(\frac{Q_i K_j^T}{\sqrt{d_{model}}}\right) \tag{S6}$$

For each element, the weighted sum across all positions yields the self-attention representation $O \in \mathbb{R}^{n \times d_{model}}$. Its calculation is as follows:

$$O_i = \sum_{j=1}^{n} A_{i,j} V_j \tag{S7}$$

This value represents the importance of each element within the entire SMILES sequence.

For the case of multi-head attention mechanism, when the number of heads $h > 1$, each element's vector representation X_i is divided into h parts. For each part, parallel linear transformations are performed to obtain X_i sets of parameter matrices: $Q_i^1, Q_i^2, \cdots, Q_i^h, K_i^1, K_i^2, \cdots, K_i^h, V_i^1, V_i^2, \cdots, V_i^h$. Attention computation is then performed for each set of parameters to obtain h sets of feature representations: $O_i^1, O_i^2, \cdots, O_i^h$. These h sets of feature representations are combined, and finally, through a parameter matrix $W_O \in \mathbb{R}^{hd_{model} \times d_{model}}$, a single linear transformation is performed to obtain the final representation. The final representation is as follows:

$$O_i = Concat(O_i^1, O_i^2, \cdots, O_i^h)W_O \tag{S8}$$

For the encoded SMILES features extracted using the attention mechanism, after being unfolded and subjected to average pooling, the final representation is given by:

$$X^{ds} = AvgPool\big(Flatten(O^{ds})\big) \tag{S9}$$

3.3 Extraction of ADRs Feature

(A) Extraction of SOC category feature

The ADR terms of each medical record are mapped to the SOC level. If there are ADRs corresponding to the SOC, they are encoded as 1; otherwise, they are encoded as 0. Through this encoding method, a 27-dimensional vector X^{as} representing the adverse reaction encoding features is obtained.

(B) Extraction of ADRs semantic features

The number of ADR terms at the PT level varies for each patient, and there are many PT-level ADR terms. Using one-hot encoding to generate features results in sparsity, making it difficult to extract features. Therefore, the PT-level ADRs involved for each patient are classified according to their primary SOC, and an attention mechanism is utilized to extract the semantic features of PT-level ADRs within each SOC level. The representation is as follows:

$$X^{ap} = AvgPool\big(Flatten(O^{ap})\big) \tag{S10}$$

4 Prediction

A prediction model based on an MLP framework is constructed for determining whether the drug is a suspected causative agent of adverse reactions, utilizing the features extracted from various sources. Data for each part has been obtained as follows: demographic features X^{pd}, MACCS molecular fingerprint feature X^{df}, molecular graph feature X^{dg}, SMILES sequence feature X^{ds}, SOC category feature X^{as}, and ADR semantic feature X^{ap}. The extracted features are concatenated for classification by SDAJM. The concatenation process is represented as follows:

$$X_{concat} = CONCAT(X^{pd}, X^{df}, X^{dg}, X^{ds}, X^{as}, X^{ap}) \tag{11}$$

A three-layer MLP classifier was constructed with ReLU activation functions (FC_{relu}) and batch normalization (BN) [9]. A fully connected layer was added at the end for aggregation. Dropout layers were included after the first two ReLU activation functions to prevent overfitting. The parameter matrix provided by the fully connected layer is denoted as W. The computation process after passing X_{concat} into the classifier can be represented as follows:

$$y = \left(FC_{relu}\left(BN\left(FC_{relu}\left(BN(X_{concat}W)\right)W\right)\right)W\right)W \tag{S12}$$

Finally, the probability that the patient's medication is a suspected drug is calculated through a Sigmoid activation function as follows:

$$p = FC_{sigmoid}(y) \tag{S13}$$

where $FC_{sigmoid}$ represents the Sigmoid activation function. If $p < 0.5$, the drug is not considered suspicious; conversely, when $p > 0.5$, the drug is regarded as a suspected drug causing ADRs.

5 Optimization of SDAJM

Hyperparameters optimization plays a crucial role in optimizing neural network models [10,11]. For optimizing SDAJM, our main optimization targets include the batch size, the number of attention heads in multi-head attention, the dropout rate for GIN and predictor's dropout layers, the learning rate, and the regularization strength. To optimize these hyperparameters, the Bayesian optimization algorithm is employed. The details of the relevant results are given in Supplementary Material S6.

Reference

1. Toropov, A.A.; Toropova, A.P.; Mukhamedzhanova, D.V.; Gutman, I. Simplified molecular input line entry system (SMILES) as an alternative for constructing quantitative structure-property relationships (QSPR). *Indian journal of chemistry. Sect. A: Inorganic, physical, theoretical & analytical* **2005**, *44*, 1545-1552.
2. Durant, J.L.; Leland, B.A.; Henry, D.R.; Nourse, J.G. Reoptimization of MDL keys for use in drug discovery. *Journal of Chemical Information and Computer Sciences* **2002**, *42*, 1273-1280.
3. Chen, Y.; Ma, T.; Yang, X.; Wang, J.; Song, B.; Zeng, X. MUFFIN: multi-scale feature fusion for drug-drug interaction prediction. *Bioinformatics* **2021**, *37*, 2651-2658, doi:10.1093/bioinformatics/btab169.
4. Cheng, X.; Cheng, M.; Yu, L.; Xiao, X. iADRGSE: A Graph-Embedding and Self-Attention Encoding for Identifying Adverse Drug Reaction in the Earlier Phase of Drug Development. *Int. J. Mol. Sci.* **2022**.
5. Xu, K.; Hu, W.; Leskovec, J.; Jegelka, S. How Powerful are Graph Neural Networks? **2019**.

6. Hamilton, W.L.; Ying, R.; Leskovec, J. Representation Learning on Graphs: Methods and Applications. **2018**.
7. Hu, W.; Liu, B.; Gomes, J.; Zitnik, M.; Liang, P.; Pande, V.; Leskovec, J. Strategies for Pre-training Graph Neural Networks. arXiv preprint arXiv:1905.12265. 2019.
8. Vaswani, A.; Shazeer, N.; Parmar, N.; Uszkoreit, J.; Jones, L.; Gomez, A.N.; Kaiser, L.; Polosukhin, I. Attention Is All You Need. In Proceedings of the 31st Conference on Neural Information Processing Systems (NIPS 2017), Long Beach, CA, USA, 4–9 December 2017.
9. Ioffe, S.; Szegedy, C. Batch Normalization: Accelerating Deep Network Training by Reducing Internal Covariate Shift. In Proceedings of the 32nd International Conference on Machine Learning, Lille, France, 7–9 July 2015.
10. Lee, C.; Yoon, J.; Schaar, M.v.d. Dynamic-DeepHit: A Deep Learning Approach for Dynamic Survival Analysis With Competing Risks Based on Longitudinal Data. *IEEE transactions on bio-medical engineering* **2020**, *67*, 122-133.
11. Liu, M.; Sun, Z.L.; Zeng, Z.; Lam, K.M. MGF6mARice: prediction of DNA N6-methyladenine sites in rice by exploiting molecular graph feature and residual block. *Brief Bioinform* **2022**, *23*, bbac082, doi:10.1093/bib/bbac082.
