# Supplementary material for: Predicting Drugs Suspected of Causing Adverse Drug Reactions Using Graph Features and Attention Mechanisms
Source: Pharmaceuticals (Basel). 2024 Jun 22;17(7):822. doi: 10.3390/ph17070822 (PMC11279999; doi:10.3390/ph17070822)
Supplement: Supplementary file 1 [file pharmaceuticals-17-00822-s001.zip › S2. Extrapolation testing and feature evaluation.pdf]

Supplementary Material S2.

Extrapolation testing and feature evaluation

1. Extrapolation testing of the model

The aim of SDAJM is to assist patients with adverse reactions in identifying suspicious drugs. However, models are often trained using historical data, which places demands on the extrapolation capability of the model. Two tests were designed to determine whether existing training data could be utilized for extrapolation by SDAJM.

Test 1 involved training and testing the model using data from different years, with independent testing conducted using the 2022 dataset. This test comprised three parts:

A. Training the model on the 2021 dataset for 10-fold cross-validation and external validation;

B. Training the model on dataset from 2020 to 2021 for 10-fold cross-validation and external validation;

C. Training the model on dataset from 2019 to 2021 for 10-fold cross-validation and external validation.

Test 2 begins by incrementally adding data from the year 2019 and validates externally using data from the remaining years. This testing is divided into four parts:

A. Training the model on the 2019 dataset and conducting 10-fold cross-validation, followed by external validation using data from 2020 to 2022.

B. Training the model on data from 2019 to 2020 and conducting 10-fold cross-validation, followed by external validation using data from 2021 to 2022.

C. Training the model on data from 2019 to 2021 and validating it externally using data from 2022.

D. Conducting 10-fold cross-validation using data from 2019 to 2022.

Figure S1 displayed the results of Test 1. Based on the results of Test 1, an improvement in the extrapolation capability of SDAJM was observed as the amount of data used increased. When the model was trained solely on data from the year 2021, the ROC-AUC for external validation on the 2022 dataset was 0.819. However, when data from both 2020 and 2021 were used for training, the ROC-AUC for external validation increased to 0.839, representing a 0.02 improvement compared to using only 2021 data. Furthermore, when data from the year 2019 was added to the training set, the ROC-AUC for external validation increased to 0.847, marking a 0.028 improvement compared to models trained solely

on 2021 dataset, and a 0.008 improvement compared to models trained on data from both 2020 and 2021 dataset.

Combining the results of Tests 1 and 2, the following conclusions can be drawn:

A. SDAJM trained on historical data demonstrates extrapolation capability and maintains stable performance on independent datasets from other years;

B. The extrapolation capability of SDAJM improves to a certain extent with the inclusion of more historical training data.

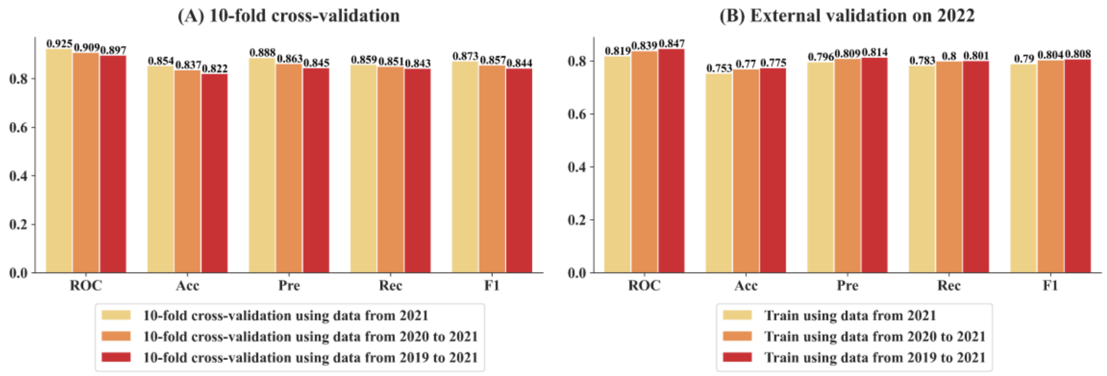

Figure S1. Extrapolation testing of the model. (**A**) Performing 10-fold cross-validation for internal validation on datasets of different lengths. (**B**) Validating models trained on datasets of different lengths externally.

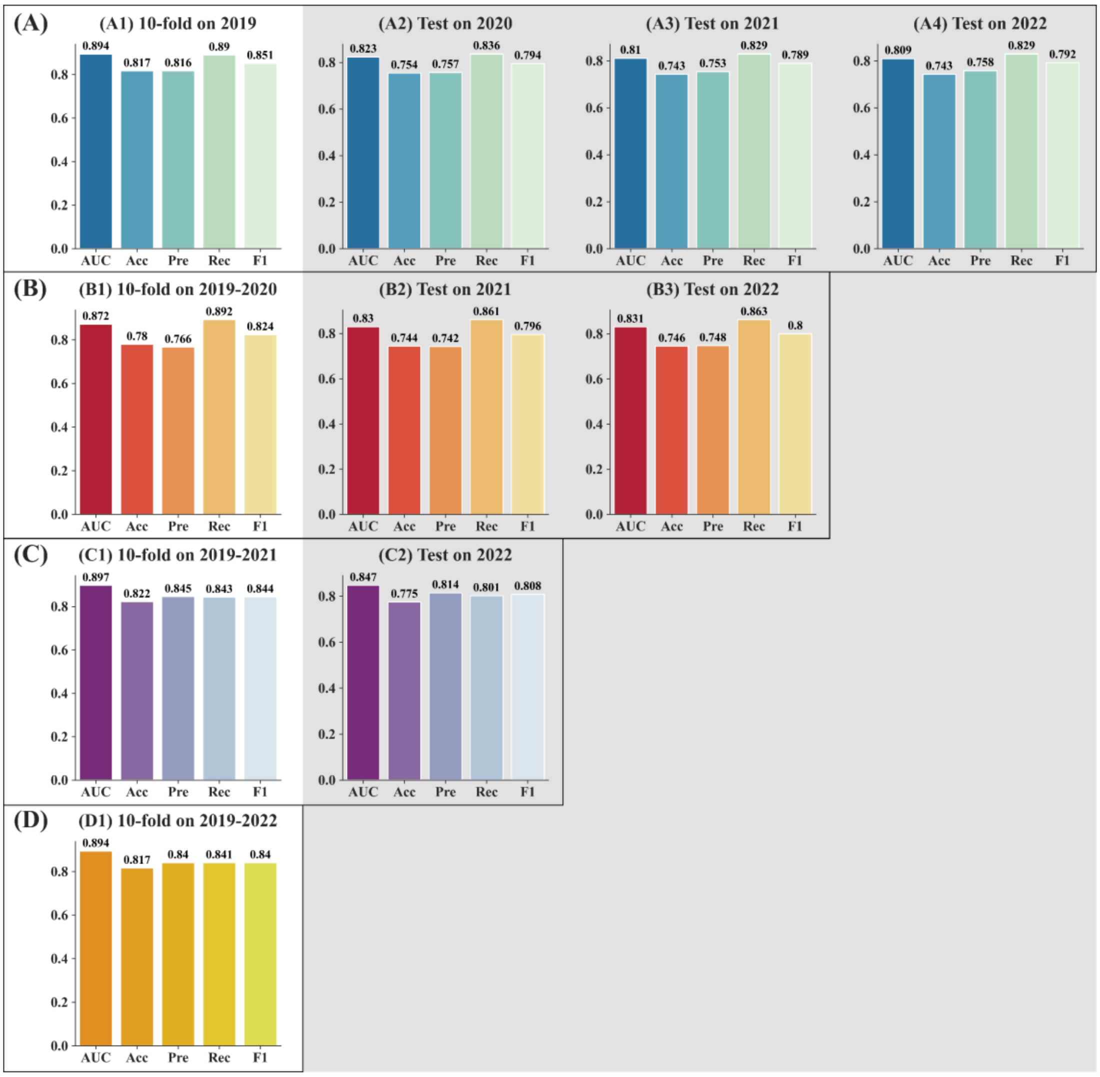

Figure S2. Extrapolation testing of the model. (**A**) Training the model on the 2019 dataset and conducting 10-fold cross-validation, followed by external validation using data from 2020 to 2022. (**B**) Training the model on data from 2019 to 2020 and conducting 10-fold cross-validation, followed by external validation using data from 2021 to 2022. (**C**) Training the model on data from 2019 to 2021 and validating it externally using data from 2022. (**D**) Conducting 10-fold cross-validation using data from 2019 to 2022.

2. Comparison of predictor

To assess the performance of the SDAJM predictor, feature extraction was conducted using SDAJM on the 2022 dataset from FAERS. The prediction component was replaced with other commonly used machine learning models, and the performance of different classifiers was compared using 10-fold cross-validation. The predictive performance of features extracted by SDAJM on various models including SDAJM predictor, Random Forest (RF), Decision Tree (DT), K-Nearest Neighbors (KNN), Logistic

Regression (LR), and Naive Bayes (NB) was compared. Table S1 shown the results of the 10-fold cross-validation, indicating excellent performance and stability of SDAJM.

Table S1 Comparison results of replacing the SDAJM predictor with other models

Model	ROC-AUC	Accuracy	Precision	Recall	F1
SDAJM	**0.9312±0.0030**	**0.8651±0.0038**	**0.8879±0.0046**	**0.8832±0.0062**	**0.8855±0.0034**
RF	0.8956±0.0247	0.8365±0.0186	0.8086±0.0473	0.8411±0.0337	0.8236±0.0290
LR	0.8612±0.0333	0.7779±0.0398	0.7406±0.0572	0.7919±0.0470	0.7646±0.0465
DT	0.7667±0.0320	0.7675±0.0317	0.7315±0.0547	0.7753±0.0518	0.7517±0.0435
NB	0.7608±0.0325	0.7523±0.0310	0.7293±0.0483	0.7255±0.0608	0.7263±0.0469
KNN	0.7515±0.0378	0.6907±0.0351	0.6516±0.0423	0.6926±0.0718	0.6701±0.0489

Note: RF represents Random Forest model. DT represents Decision Tree model. KNN represents K-Nearest Neighbors model. LR represents Logistic Regression model. NB represents Naive Bayes model.

3. Feature evaluation

The performance of various components of SDAJM was compared. To reduce computation time, 10-fold cross-validation was conducted on the FAERS 2022 dataset only. Additionally, the performance of replacing attention components of SDAJM with other residual networks (ResNet) [39, 40] and convolutional neural networks (CNN) was compared.

Tables S2 and S3 shown the results of feature evaluation. It was observed that using only demographic features along with ADR features or only demographic features along with drug features led to poorer performance compared to the complete model. The ROC-AUC decreased by 0.0865 and 0.0727, respectively, when compared to the complete model. However, the model's performance improved when all three sets of features were used together, showing better average performance compared to models using only two sets of features.

The use of attention mechanism on drug SMILES feature played a crucial role in SDAJM, resulting in a 0.06 increase in ROC-AUC. When comparing feature extraction methods using ResNet and CNN, the attention mechanism demonstrated superior performance, leading to ROC-AUC increases of 0.0206 and 0.0283, respectively. The results of feature comparison indicate:

A. The drug SMILES feature and ADRs semantic feature, which utilize the attention mechanism, play crucial roles in SDAJM.

B. The feature extraction method using the attention mechanism is more effective in SDAJM compared to ResNet and CNN.

Table S2 The comparative results of the features used in SDAJM

**Features Set**						**Evaluation metrics**				
X^{pd}	X^{df}	X^{dg}	X^{ds}	X^{as}	X^{ap}	**ROC-AUC**	**Accuracy**	**Precision**	**Recall**	**F1**
√	√	√	√			0.8623	0.7745	0.8314	0.7769	0.8031
√				√	√	0.8485	0.7546	0.8642	0.6951	0.7703
	√	√	√	√	√	0.9254	0.8586	0.8734	**0.8904**	0.8818
√		√		√	√	0.8552	0.7706	0.8533	0.7400	0.7925
√	√	√		√	√	0.8750	0.7864	0.8743	0.7469	0.8055
√	√	√	√	√	√	**0.9350**	**0.8679**	**0.8951**	0.8803	**0.8876**

Table S3 The comparative results of the feature extraction methods used in SDAJM

**Model**	**ROC-AUC**	**Accuracy**	**Precision**	**Recall**	**F1**
SDAJM	**0.9350**	**0.8679**	**0.8951**	**0.8803**	**0.8876**
SDAJM -ResNet	0.9144	0.8747	0.8798	0.8686	0.8741
SDAJM -CNN	0.9067	0.8649	0.8720	0.8560	0.8639

Note: SDAJM-ResNet replaces the attention components with ResNet. SDAJM-CNN replaces the attention components with CNN.
